# Editorial: Effects and mechanisms of bariatric surgery in relieving obesity and its complications

**DOI:** 10.3389/fendo.2025.1563980

**Published:** 2025-02-07

**Authors:** Lidia Castagneto-Gissey, Yayun Wang, Kaixiong Tao

**Affiliations:** ^1^ Department of Surgery, Sapienza University of Rome, Rome, Italy; ^2^ Department of Gastroenterology, Air Force Medical University, Xi’an, China; ^3^ Wuhan Union Hospital, Tongji Medical College, Huazhong University of Science and Technology, Wuhan, China

**Keywords:** obesity, bariatric surgery, gut microbiota, gerd, genetic variability, PCOS, MASH

The global prevalence of obesity has reached alarming proportions, with its associated comorbidities imposing a significant workload on healthcare systems worldwide (1). Obesity contributes to a spectrum of metabolic, cardiovascular, and inflammatory conditions, including type 2 diabetes (T2D), metabolic dysfunction-associated steatohepatitis (MASH), and renal dysfunction, while also exacerbating issues such as periodontitis and irregular menstruation (2). Bariatric surgery has emerged as a transformative intervention for managing morbid obesity, offering not only sustained weight loss but also profound metabolic benefits (3–5). The studies featured in this Research Topic illuminate the multifaceted effects and mechanisms of bariatric surgery, particularly focusing on Roux-en-Y gastric bypass (RYGB) and sleeve gastrectomy (SG), in addressing obesity and its complications. This Research Topic spans through the key findings from these research papers, highlighting the evolving understanding of bariatric surgery’s therapeutic potential.

Weight loss remains a cornerstone of bariatric surgery’s benefits, with studies underscoring its pivotal role in mediating metabolic and hormonal improvements. For instance, Tian et al. demonstrated that total weight loss (TWL%) following bariatric surgery is closely correlated with reductions in thyroid-stimulating hormone (TSH) levels, suggesting enhanced thyroid function in euthyroid patients with obesity. Interestingly, this relationship was independent of baseline factors such as age, sex, or comorbidities, emphasizing the universal metabolic benefits of significant weight loss.

Similarly, Zhao et al. investigated the effects of SG on women with polycystic ovary syndrome (PCOS) and found that weight loss—rather than preoperative body mass index (BMI)—was a critical determinant of menstrual regularity. With 79.03% of participants achieving remission of irregular menstruation within a year, this study highlights the potential of bariatric surgery to address reproductive dysfunctions associated with obesity. These findings underscore the importance of targeting substantial weight loss to maximize health outcomes, regardless of baseline BMI.

Gender-specific differences in predictors of weight loss after SG were highlighted in the study by Shu et al. Both men and women achieved comparable weight loss outcomes when baseline characteristics were matched. However, distinct predictors emerged: in men, baseline BMI and insulin dynamics were significant, while in women, age, thyroid function, and mental health measures played a more substantial role. These insights advocate for personalized preoperative evaluations to optimize weight-loss outcomes across genders.

The role of gut microbiota in mediating the metabolic benefits of bariatric surgery has garnered significant attention. Liu C. et al. employed a multi-omics approach to investigate changes in gut microbiota and metabolism following SG. They reported increased microbial diversity and enrichment of beneficial bacteria such as Alistipes and Parabacteroides. These microbial changes were correlated with enhanced lipid metabolism, as evidenced by elevated levels of free fatty acids and bile acids. Similarly, the study by Li et al. on duodenojejunal bypass (DJB) in obese T2D mice demonstrated that proximal gut microbiota play a dominant role in improving glucose metabolism and reducing inflammation. Both studies underscore the essential role of intestinal remodeling in achieving metabolic improvements post-surgery.

Bariatric surgery is not without complications, and gastroesophageal reflux disease (GERD) is a prominent concern, particularly after SG (6, 7). Liu G. et al. provided a comprehensive review of strategies to manage GERD in these patients, emphasizing the efficacy of RYGB as the gold standard for refractory cases. Innovative approaches such as magnetic sphincter augmentation and antireflux mucosectomy were also explored, offering minimally invasive alternatives for GERD management.

Periodontal health, often overlooked in obese populations, was addressed by Bi et al. Their study found significant improvements in periodontal parameters such as plaque index (PLI) and bleeding index (BI) following SG, particularly in patients with T2D. Reduced systemic inflammation, as evidenced by declines in hs-CRP and IL-6 levels, was likely a key contributor to these improvements. However, persistent disparities in pocket depth between diabetic and non-diabetic groups highlight the need for continued oral health monitoring in this population.

Chen et al. explored the molecular mechanisms underlying MASH improvement post-bariatric surgery, identifying key genes such as FASN, SCD1, and HMGCS1 that regulate lipid metabolism and inflammation. Downregulation of these genes was associated with reduced liver steatosis and enhanced metabolic health, offering potential therapeutic targets for non-surgical interventions.

Another metabolic dimension, glycine deficiency, was investigated by Tan et al. Obesity-induced glycine deficiency impairs the glycine conjugation pathway, which is crucial for detoxification and metabolite clearance. Their findings demonstrated that bariatric surgery restores glycine synthesis and improves detoxification, highlighting an additional mechanism through which weight loss enhances systemic health.

Obesity-related renal dysfunction, a growing concern, was examined by Popa et al., who emphasized gender-specific influences of visceral adiposity on renal filtration. In females, visceral adipose tissue (VAT) mass and its associated metabolic effects played a dominant role in renal dysfunction and recovery. By contrast, in males, weight loss and lean mass changes were more influential. Postoperatively, significant improvements in estimated glomerular filtration rate (eGFR) were observed across all renal function categories, reinforcing the reno-protective effects of bariatric surgery.

The interplay between genetics and weight loss outcomes was highlighted by Wang et al., who identified the CAMKK2 variant as a novel monogenic obesity variant (MOV). Using whole-exome sequencing and postoperative data, they demonstrated that carriers of the CAMKK2 mutation experienced less pronounced weight loss and metabolic improvements compared to non-carriers. This study underscores the importance of integrating genetic insights into personalized obesity treatment strategies.

The studies in this Research Topic collectively highlight the multifactorial mechanisms underlying bariatric surgery’s success in addressing obesity and its complications. From gut microbiota remodeling and molecular pathway modulation to gender-specific influences and genetic predispositions, these findings emphasize the importance of a multidisciplinary approach in understanding and optimizing surgical outcomes.

While the benefits of bariatric surgery are well-documented, challenges remain in ensuring equitable access, managing complications, and refining surgical techniques. Further research is needed to develop standardized guidelines for managing postoperative complications such as GERD and nutritional deficiencies; explore long-term outcomes in diverse populations, with particular attention to sex and genetic variability; leverage multi-omics approaches to identify novel biomarkers and therapeutic targets for obesity-related diseases; enhance interdisciplinary care, integrating endocrinology, gastroenterology, and behavioral health to address the holistic needs of patients.

The insights presented in this Research Topic advance our understanding of the underlying mechanisms, paving the way for personalized and effective treatment strategies. By continuing to integrate clinical research with molecular and genetic insights, we can further harness the potential of bariatric surgery to improve the lives of individuals living with obesity.
